# Measuring the level of implementation of advance care planning – a fidelity-based cross-sectional study

**DOI:** 10.3389/frhs.2025.1629242

**Published:** 2025-10-29

**Authors:** Siri Færden Westbye, Maria Romøren, Marc Ahmed, Karin Berg Hermansen, Linn Brøderud, Astrid Klopstad Wahl, Trygve Johannes Lereim Sævareid, Lisbeth Thoresen, Reidar Pedersen

**Affiliations:** ^1^ Centre for Medical Ethics, Institute for Health and Society, Faculty of Medicine, University of Oslo, Oslo, Norway; ^2^ Department of Geriatric Medicine, Oslo University Hospital, Oslo, Norway; ^3^ Department of Health Sciences, Norwegian University of Science and Technology, Ålesund, Norway; ^4^ Department of Public Health and Interdisciplinary Health Sciences, Institute of Health and Society, Faculty of Medicine, University of Oslo, Oslo, Norway

**Keywords:** advance care planning, implementation science, patient centered, older patients, health systems

## Abstract

**Introduction:**

Advance care planning (ACP) is supported by evidence, legal and ethical principles and ACP guidelines. However, this complex intervention is often poorly implemented. This article presents a novel fidelity scale to measure the implementation of ACP and reports the preliminary testing of the scale at baseline in a larger trial aiming at implementing ACP in hospitals in Norway.

**Method:**

The research design conducted was a cross-sectional measurement of fidelity to guidelines, conducted in 12 geriatric hospital units in Norway recruited using purposive sampling. The sample size for the larger trial was determined, based on prior research, to be at least four clusters in each arm. Due to the possibility of unit drop-out during the project period and to secure sufficient power, we included six units in the intervention arm and six in the control arm. For the baseline measurement we developed an ACP fidelity scale including three subscales: implementation, quality and penetration rate, each with 4–11 items. We ensured face and content validity through input from relevant theories and research, experts and users. Items were scored from 1 to 5, with 1 indicating no implementation and 5 indicating full implementation. Data was analyzed using descriptive statistics, Cronbach's alpha and calculation of interrater reliability for the scales. Further psychometric testing could not be conducted at this stage due to the lack of variance in the items.

**Results:**

The mean score for the implementation subscale was 1.213, with a median of 1, a standard deviation (SD) of 0.08, a standard error (SE) of 0.01, a variance of 0.01, and a range of 0.28 (minimum 1.14 and maximum 1.42). The scores in the subscale showed that none of the units had recommended implementation measures. Only a few professionals reported they had heard of ACP, but not the whole staff. Cronbach's alpha could not be estimated due to the lack of variation in the scores for this subscale. On the quality subscale, which assesses whether ACP is practiced in accordance with practice guidelines, the mean score was 1.11, the median was 1, the SD was 0.48, the SE was 0.06, the variance was 0.13, and the range was 1.27 (minimum 1 and maximum 2.27). The scores in this subscale showed that ACP was practiced sporadically by the palliative care team in only one unit, while the other staff did not engage in this practice at all. Cronbach's alpha for the subscale on quality was 0.887 (11 items) showing an acceptable internal consistency. For the penetration rate subscale, which measures how widespread the practice is, the mean score was 1.08, the median was 1, the SD was 0.28, the SE was 0.05, the variance was 0.08, and the range was from a minimum of 1 to a maximum of 2. Among the total number of admitted geriatric patients, only 10% had received ACP in only one of the 12 units. Also, for this subscale, the model for Cronbach's alpha could not be applied. There was little variation in the low measurements, thus the interrater reliability was high, reflected in the Intraclass Correlation Coefficient (ICC). The ICC was 0,916 [−0,721,0.976] for the implementation subscale, 1.00 for the quality subscale, and 1.00 for the penetration rate subscale.

**Conclusion:**

Our findings indicate very low implementation of ACP in acute geriatric hospital units in Norway. The newly developed ACP fidelity scale has the potential to serve as an important tool for improving the quality of healthcare services for older patients. However, more data is needed to validate the psychometric properties of the scale. Our study should be considered as a preliminary study, and the scale should be used with caution as long as its properties are not well validated.

**Clinical Trial Registration:**

ClinicalTrials.gov, NCT05681585.

## Introduction

ACP is defined as a process that supports patients in understanding and communicating their personal values, life goals, and preferences regarding present and future medical care to ensure that people receive medical care consistent with these preferences (1). Advance care planning (ACP) is increasingly recommended in clinical practice guidelines beyond palliation (2, 3), also in Norway (4, 5). However the implementation of ACP is patchy and numerous barriers to implementation have been reported (6–9). This is despite the recent evidence synthesis showing that ACP has the potential to support the involvement of older patients and their next of kin and improve communication (10, 11) and is today a global problem (8).

To improve the implementation of ACP, a first step is to measure the degree of implementation of ACP. Within implementation science various “fidelity”-scales to measure implementation of evidence-based practices (EBP) have been developed (12). Such scales can be used to measure the degree of implementation of an EBP and monitor the effects of implementation programs (12).

“Fidelity” can be narrowly defined as adherence to evidence-based guidelines focusing on how to do the clinical intervention, to ensure adequate quality when the intervention is applied. However, “fidelity” can also be defined more broadly, to include also an assessment of implementation interventions, i.e., any measures or strategies used to ensure that the clinical intervention is used in line with EBP-guidelines, and to what extent the clinical intervention is used (often called “penetration”) (13). Most fidelity studies focus on the narrower definition of fidelity (14). This also applies to the relatively few ACP implementation studies assessing fidelity that we have been able to identify (15, 16).

Both ACP as a clinical intervention, and ACP-implementation measures and strategies can be defined as “complex interventions”, e.g., interventions that require a range of behaviors required of those delivering or receiving the intervention at different levels (17). Broader approaches in implementation studies and fidelity measurements are particularly important when it comes to complex interventions, because an intervention or treatment will not be effective if it is not implemented well, e.g., perfect ACP-quality is of limited value if ACP is very rarely done (18).

However, we were not able to identify any measurements developed to comprehensively assess the implementation of ACP. Thus, we decided to develop a fidelity scale based on the broad definition of fidelity.

This was done as part of a larger study aiming at improving the implementation of ACP in the routine care of acutely admitted home-dwelling geriatric patients in Norway (19), where the scale was used to assess the implementation of ACP at baseline in the participating units.

The aim of this article is both to present the development of the new ACP fidelity scale, and to report the results of the baseline assessment as/including a preliminary validation of the scale.

## Material and methods

This article corresponds to the 'Strengthening the reporting of observational studies in epidemiology (STROBE) statement' (20) (Please insert Supplementary File 1 here).

### Study design, setting and participating units

This study examines the current level of ACP at baseline, prior to conducting a trial aimed at implementing ACP in the routine care of acutely admitted patients in acute geriatric units (19). We have chosen a more general geriatric hospital setting because ACP has become increasingly relevant beyond palliation and oncology (2) and because this context has been little studied (21). The literature on ACP mostly covers the context of end-of-life care and/ or nursing homes. But the expert consensus definition for ACP recommends it across the life continuum (1) and that it should ideally start early in adulthood (1). A preceding study in Norwegian nursing homes found ACP may profit from commencing before nursing home admission due to cognitive impairment (22). In this project we therefore included home dwelling patients, that were acutely admitted to hospital. Previous research shows that the hospital setting can increase the relevance of ACP (23). Furthermore, we included patients living with frailty because they are a particularly “in need” group, given their increased risk of morbidity and mortality (21). The reason for selecting geriatric units was also that we found a high level of competence in communication with older patients in these units, as well as a motivation and interest for ACP.

The study is based on a cluster-randomized design and included 12 hospital units (19). A cluster was defined as an acute geriatric unit or an internal medicine unit with a geriatrician among staff. In Norway, geriatrics is a “branch” of internal medicine. This implies that one has to specialize in general internal medicine before one can become a specialist in geriatric medicine, that is a specialist in diseases in old age. We thus included acute geriatric units, and internal medicine units with a geriatrician among staff when this was the only offer to geriatric patients. 12 out of 15 eligible units in the Southeast Norway Regional Health Authority agreed to participate, covering a catchment area of over two million inhabitants. The main reason for the non-participation of the remaining units was the lack of time and personnel.

After randomization, the intervention arm received 18 months of training and support in implementing ACP guidelines and national recommendations. To increase implementation, the control arm received a shorter version of the same program after the 18 months period. Fidelity was measured in the units at baseline and two follow ups: at 7–10 months and 18 months. For the study as a whole, the sample size was calculated to be at least four clusters in each arm. Due to the possibility of unit drop-out during the project period and to secure sufficient power, we included six units in the intervention arm and six in the control arm (19). Table 1 summarizes the characteristics of all clinical units, and the variation in the organization. The lack of time and resources, including adequate staffing, is a significant barrier highlighted in the literature (6, 24, 25). In our sample the number of patients per Full-Time Equivalent (FTE) staff varied considerably with a range of 3.18 (minimum 1.77 and maximum 4.95).

**Table 1:** Description of the 12 clinical sites included in the cluster randomized trial.

Site	Type of unit^a^	Catchment population	The maximum of patients in the units	Reported percentage of the maximum number of patients who have participated in ACP in the last six months	Mean length of stay for acute geriatric patients (days)	Full-time equivalent staff (FTE)	Patients per FTE
1	IMU	30 267	10	10%	3,48	17,7	1,77
2	AGU	50 000	6	0%	4,55	23	3,83
3	AGU	60 000	5	0%	3,3	30,7	1,33
4	AGU	120 000	9	0%	4	40	4,44
5	AGU	150 000	16	0%	4,1	42,7	2,67
6	AGU	155 000	4	0%	2,5	19,8	4,95
7	AGU	168 000	8	0%	4	39,4	4,9
8	AGU	180 000	8	0%	3,6	33,7	4,21
9	AGU	200 000	20	0%	4,9	36	1,8
10	AGU	230 000	9	0%	4	16,5	1,83
11	AGU	323 453	18	0%	4	47,2	2,6
12	AGU	594 000	23	0%	6	53,5	2,32

^a^ AGU: Acute geriatric unit, IMU: Internal medical unit with geriatrician among staff.

### Instruments

In the present survey, we developed three subscales with 4–11 items (22 items in the total scale). The scale was written in Norwegian, and the study was conducted in Norwegian, but we have translated the scale into English to share this instrument with clinicians and researchers (please see Supplementary File 2).

**Figure 1:**
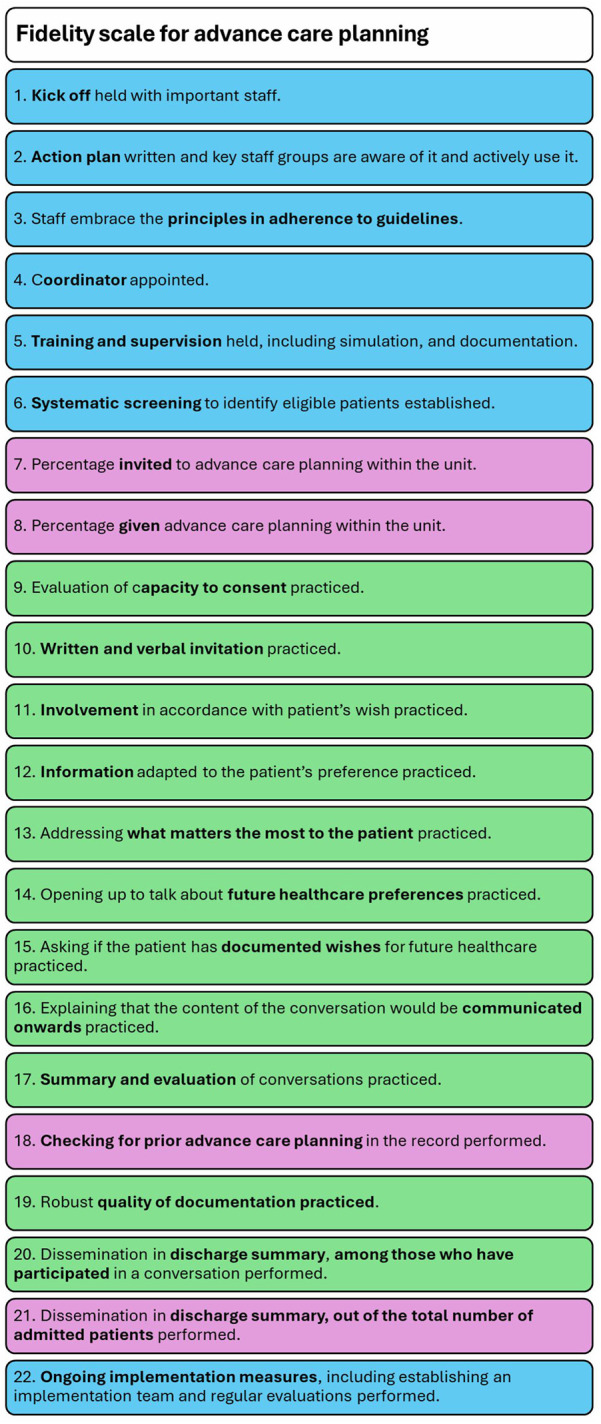
Schematic overview of fidelity scale for advance care planning (implementation measure items are marked in blue, quality of practice items in green, and penetration rate items in purple).

In each scale, items are scored on a scale from 1 to 5, with 1 indicating no implementation and 5 indicating full implementation. The scores of the items are added together and divided by the total number of items in the respective subscales, and the overall scale to determine the average scores. The purpose of the scales was to operationalize the guidelines and recommendations for ACP practice and implementation support in healthcare. The order of items is chronological as a model for action, and to ensure a logical and easily understandable order for the researchers and the units being evaluated, as well as for other users of the scale (26). A schematic overview of the scale can be seen in Figure 1.

The study protocol describes how the project group developed the ACP interventions by selecting recommendations from the guidelines and synthesizing them into key elements (19).

**Figure 2:**
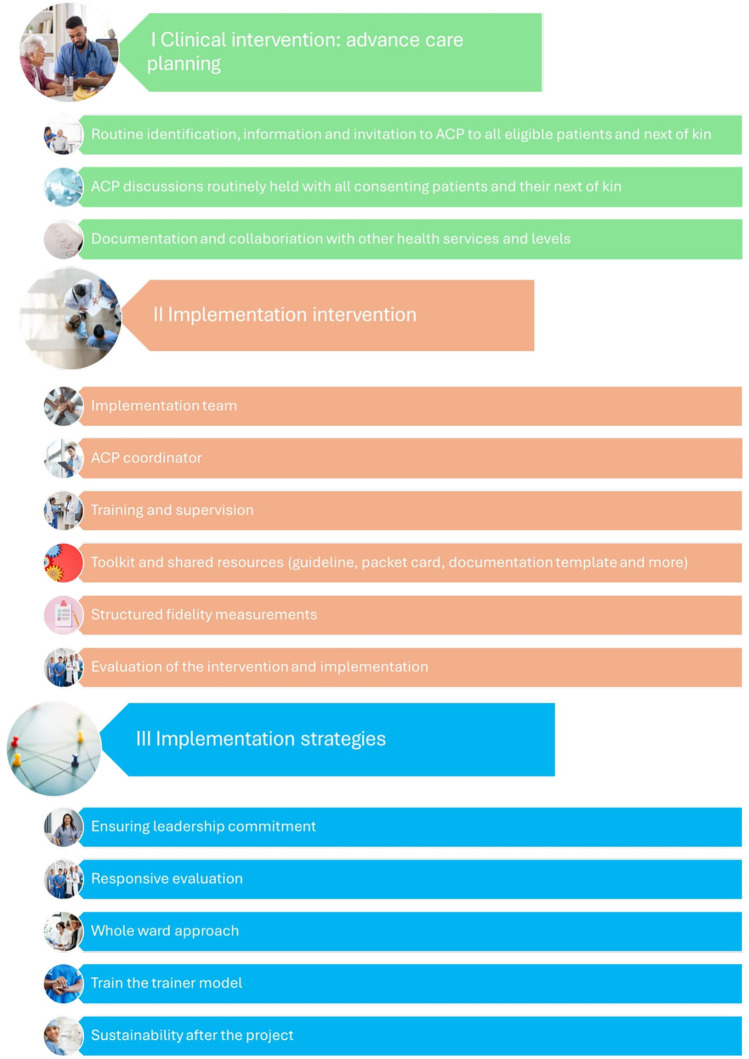
ACP interventions in the larger implementation project. (We have changed the terminology in the project after the publication of the protocol article, so that we now define implementation intervention as both implementation measures and implementation strategies. Together with the clinical intervention, this constitutes the entire ACP intervention package in the project. The new terminology is to be presented in a forthcoming article).

In parallel to this process, we developed the items that would cover these key elements of the ACP interventions. A description of the ACP interventions can be found in the study protocol (19). An overview of the interventions can be seen in Figure 2.

In developing the scales, we sought to include items that measure both practice adherence to the guidelines and the implementation measures needed to sustain the practice, as well as the penetration rate. By penetration rate, we mean the percentage of eligible patients and/or next of kin who receive a particular invitation, treatment, service, or practice (27). Thus, the scale emphasizes the importance of reaching a large number of patients while also practicing ACP accurately. Thus, the three subscales measure 1) the quality of the intervention, 2) an assessment of the implementation measures and 3) the penetration rate in the participating units.

An overview of the evidence base for the items can be found in Supplementary File 4.

### Methodology

The project group followed the standardized procedure for scale development described by Bond and colleagues (12). For the subscale measuring the quality of ACP practiced, we constructed the items based on the Delphi panel recommendations (1–3), which are also recommended as the most rigorous source in the development of new scales (12). The second most important base was Up to Date's summary of the practical steps recommended in an ACP conversation (28), which complements the Delphi panel's consensus definitions (1, 2). We have included that the patient's decision-making capacity should be assessed as one of the steps in ACP (28). This is recommended in the national guidelines because the role of next of kin becomes more important in a situation where the patient's capacity to consent is weakened - either as a support for the patient or as a representative of the patient when the patient's competence has ceased (5). This is in line with the most recent consensus definition of patients with dementia (3). Under Norwegian law, next of kin do not have the authority to make medical decisions on behalf of the patient; however, healthcare providers are required to consult them regarding the patient's wishes. Serious interventions may only be performed if they align with the patient's best interests and if there is reason to believe that the patient would have consented had they been able to do so (29).

The third source we used to develop the items, as recommended by Bond, was systematic reviews on ACP (12). An overview of the literature search for the reviews can be found in Supplementary File 3. Randomized controlled trials that were included in these reviews were trials that provided a description of the implementation programs or a description of the intervention or the training program for healthcare professionals. A challenge was that ACP interventions vary widely in the literature (8). The interventions used were also poorly described and were often licensed programs that are not openly accessible. However, some recurring steps would be the identification of patients approaching the end-of-life, assessment or exploration of the patient's needs and personal values, conversations about different care options and choices, as well as final decisions and ensuring a coordinated documentation of ACP which we made sure were reflected in the items on quality (21, 30, 31).

To adapt the quality subscale to the Norwegian context, the most useful source was an interview guide for ACP that was tested in a cluster randomized trial in nursing homes (32). The trial used a whole-ward approach, which is also a strategy used in this study. The strategy aims at involving the entire staff in the ACP process, facilitating both formal conversations where the patient and their next of kin are invited to engage, as well as informal discussions where patients can initiate conversations with staff about what matters to them, such as during care or other daily activities. We also ensured that the scale was in line with the recent Norwegian recommendations on ACP (5). Finally, we looked at a procedure for ACP developed at Oslo University Hospital (33). The procedure is based on the Gold Standard Framework, which is one of several ACP models (34).

The items in the subscales measuring implementation support and penetration rate were generated based on previous scales validated and tested for reliability in the project to improve family engagement (13), as no established measures of ACP implementation or quality exist. We have also included a review article presenting the Gold Standard Framework implementation model (35). In addition, we have included the known barriers and facilitators to ACP implementation to systematize the areas that would be relevant in the implementation process in the implementation support subscale. An overview of the literature search for barriers and facilitators can be found in Supplementary File 3.

The next step involved content validation, which included gathering feedback from experts and users to inform revisions of the items in group meetings. Content validity refers to the extent to which the items in a test are reasonably representative of the entire domain that the test attempts to measure (36). In response to feedback, in some cases tasks were tailored to local circumstances, which is recommended when implementing complex interventions (17). An important example is how ACP should be documented and communicated in a context where documentation systems differ from hospital to hospital, and where the primary healthcare system lacks a unified system that is shared with specialist health services. With input from experts and clinicians, we concluded that the most important themes discussed during the conversation, along with any specific preferences regarding treatment and care, should be included in the discharge summary. This document is read by the general practitioner or nursing home physician. Consequently, we included this as an item in the penetration rate, to measure not only how many patients received ACP, but also how many of them had ACP documented appropriately to be effective beyond that hospital stay. We also considered content validity during piloting which consisted in testing the version of the scale we had agreed on during internal validation with staff groups that would be interviewed, e.g., a geriatrician, a head of department, and a nurse. Here we also sought to ensure face validity, i.e., the appropriateness, meaningfulness, or relevance of the test as it appears to the people answering the test (37). User acceptability, as recommended by Bond and colleagues (12), was further assessed during baseline measurements. An exception to the Bond and colleagues' methodology is that the scale could only be tested to a limited extent in this study due to time constraints. Some items were therefore modified after the baseline data was collected. The scale was adjusted both at the 7–10-month follow-up and 18-month follow up, which resulted in some minor changes to the scale. We present the changes made and the rationale behind them in the amended version of the scale in Supplementary File 2 (please see Supplementary File 2). In this article we present the amended version of the scale that was used in the larger study implementing ACP (please see Supplementary File 2).

We chose to use the scale in the larger study before it had been more thoroughly validated because we had limited time and resources available, because we had had good experience with a similar scale in the previous study investigating family involvement in people with psychotic disorders (38), and because we had conducted a relatively thorough development process in advance. However, we also consider that the results need to be interpreted with caution until further validation is available, and for the time being should be regarded as the results of a preliminary validation. At the same time, our experience and reliability tests gave us no reason to make any major changes or not to continue with this scale in the larger study.

### Data collection

The clinical units were recruited from fall 2021 to February 2022. Baseline measurements took place between May and July 2022. Fidelity as a method can use different data sources, such as observations, interviews with staff, or a review of medical records and program documents (12). In this fidelity study trained fidelity assessors visited each unit and measured fidelity by conducting structured interviews with the heads of the department, clinicians and any local resource persons such as the palliative care team. Some of the measurements were conducted digitally for feasibility reasons. We considered this acceptable because none of the units practiced ACP in a systematic way.

We also asked whether the units had any written or online material on ACP. Only one unit had a written procedure for ACP. The head of the department (department or section head) was interviewed individually, while clinicians and resource persons (if applicable) were interviewed in separate groups of 1–5 people. In total, 2–4 interviews lasting 1–1.5 h were conducted at each site. We also collected organizational data (Table 1).

At each site, the two fidelity assessors first rated all items independently and then arrived at a common rating for each item. The assessors and the composition of assessors varied from site to site. The raters were selected from a pool of eight researchers who also worked in healthcare, but none of them worked at the clinical units.

The assessors prepared a detailed report for each site, which was attached to the score results. The results and reports were sent to the units in the intervention arm after randomization so that they could correct any misunderstandings and adjust the results if necessary. However, none of the units provided feedback that led to an adjustment of the scores. The units in the control arm did not receive their assessments or reports so as not to influence them during the intervention phase.

### Data analysis

We examined item distributions for all three subscales, including means, median, standard deviation (SD), standard error (SE), ranges, minimum and maximum values and the number of units that achieved low, adequate, and full implementation of the various items. All data analyzes were conducted using SPSS version 28.

Based on the organizational data from the clinical units and the scores, we also calculated the percentage of patients admitted to the ward during the last six months who had received ACP.

## Results

The distribution of items and interrater reliability are presented in Table 2.

**Table 2:** Fidelity baseline results, distributions, interrater reliability and percentage agreement for the fidelity scale, after testing in the 12 hospital units.

Item number	Item description	Mean	Median	SD	SE	Variance	Range	Minimum	Maximum	Numbers of sites achieving score			Agreement (%)	ICC (confidence intervals)
	Implementation subscale									1	2–3	4–5		
1	Meeting	1	1	0	0	0	0	1	1	12	0	0	100	1.000
2	Action plan	1	1	0	0	0	0	1	1	12	0	0	100	1.000
3	Program philosophy	2.25	2	0.11	0.11	0.42	3	1	4	1	11	0	66.67	0.784 [0.284,0.937]
4	Coordinator	1.33	1	0.48	0.08	0.23	1	1	2	8	4	0	100	1.000
5	Training and supervision	1	1	0	0	0	0	1	1	12	0	0	100	1.000
6	Systematic identification of eligible patients	1	1	0	0	0	0	1	1	12	0	0	100	1.000
22	Continuous implementation measures	1	1	0	0	0	0	1	1	12	0	0	100	1.000
	Subtotal for implementation measures	1.23	1	0.08	0.01	0.01	0.28	1.14	1.42	12	0	0	—	0.916 [−0,721,0.976]
	Cronbach's alpha for implementation subscale	N/A												
	Quality subscale													
9	Assessing decision-making capacity	1	1	0	0	0	0	1	1	12	0	0	100	1.000
10	Verbal and written invitation.	1	1	0	0	0	0	1	1	12	0	0	100	1.000
11	Level of involvement	1.08	1	0.28	0.47	0.79	1	1	2	11	1	0	100	1.000
12	Provide information	1	1	0	0	0	0	1	1	12	0	0	100	1.000
13	What is important to the patient	1.08	1	0.28	0.47	0.79	1	1	2	11	1	0	100	1.000
14	Elicit preferences regarding future and current treatment and care	1.08	1	0.28	0.47	0.79	1	1	2	11	1	0	100	1.000
15	Ask the patient if they have written down any previous wishes regarding healthcare	1 (0)	1	0	0	0	0	1	1	12	0	0	100	1.000
16	Permission to pass on information to other health personnel	1.08	1	0.28	0.47	0.79	1	1	2	11	1	0	100	1.000
17	Summing up and evaluating the ACP-discussion	1.25	1	0,84	0.14	0.71	3	1	4	11	0	1	100	1.000
19	Quality of documentation	1.25	1	0,48	0.14	0.71	3	1	4	11	0	1	100	1.000
20	Dissemination of ACP in discharge summary	1.33	1	1,12	0.19	1.26	4	1	5	11	0	1	100	1.000
	Subtotal for quality	1.11	1	0,36	0.06	0.13	1.27	1	2.27	11	1	0	—	1.000
	Cronbach's alpha for quality subscale	0.887												
	Penetration rate subscale													
7	Proportion invited	1 (0)	1	0	0	0	0	1	1	12	0	0	100	1.000
8	Proportion given ACP (penetration rate in the unit)	1 (0)	1	0	0	0	0	1	1	12	0	0	100	1.000
18	Checking in the patient's record if ACP has been done previously	1,33	1	1,12	0,19	1,26	4	1	5	11	0	1	100	1.000
21	ACP in discharge summary	1 (0)	1	0	0	0	0	1	1	12	0	0	100	1.000
	Subtotal for penetration rate	1.08	1	0,28	0.05	0.08	1	1	2	12	0	0	—	1.000
	Cronbach's alpha for penentration rate subscale	N/A												
	Scale total	1.13	1	1.16	0.03	0.26	0.60	1.05	1.64	12	0	0	—	0.983 [0,945;0,995]

For the implementation subscale, the mean score was 1.213, with a median of 1, a SD of 0.08, a SE of 0.01, a variance of 0.01, and a range of 0.28 (minimum 1.14 and maximum 1.42). This reflected that in four of the units, an ACP coordinator had been appointed prior to their involvement in the project, but no kick-offs had been held with important staff, and there were no action plans for implementing ACP in the units or leadership commitment concerning ACP. As a result, none of the units had successfully integrated ACP into their organizational practices.

Regarding practical training, no units offered this to staff, apart from one digital theoretical course that had been held for physicians. In terms of supervision, two units had routines in place to discuss ethical issues for the nurses, but not for physicians or other staff. A minority of staff in five of the units were familiar with the principles of ACP, primarily through English-language literature or a personal interest in palliative care. However, the key principles of ACP according to guidelines was not fully understood by staff. Furthermore, none of the units had systems in place to identify patients that could be eligible for ACP. Cronbach's alpha could not be estimated because the items lacked sufficient variance.

For the quality subscale, the mean score was 1.11, the median was 1, the SD was 0.48, the SE was 0.06, the variance was 0.13, and the range was 1.27 (minimum 1 and maximum 2.27). The assessment of decision-making capacity specifically for ACP was not practiced in any of the units. Involvement of patients and their next of kin was also patchy due to the lack of time, and when they did take place, conversations were almost only about the limitation of life-prolonging treatment and not ACP. In one of the units ACP was practiced sporadically by the palliative care team. They asked about the level of involvement, what was important to the patient, and identified preferences and asked for the permission to share them, as well as also documented ACP in the discharge summary, covering a considerable amount of principles. However, in none of the units ACP was fully practiced as recommended or a systematic practice part of routine care. Cronbach's alpha for the subscale on quality was 0.887 (11 items) showing an acceptable internal consistency.

In the subscale measuring the penetration rate of ACP, the mean score was 1.08, the median was 1, the SD was 0.28, the SE was 0.05, the variance was 0.08, and the range was from a minimum of 1 to a maximum of 2. This shows that in the previously mentioned unit, the palliative care team checked if the patient had participated in ACP previously in the patient record. Notably, this was also the only unit reporting that they had offered ACP to patients during the last six months; however, only 10% of their admitted patients had received this offer (please see Table 1). In contrast, the majority of staff across the other 11 units did not engage in ACP. Cronbach's alpha could not be calculated due to insufficient variance among the items.

We also calculated the percentage of exact agreement for each item and examined the interrater reliability reflected in the Intraclass Correlation Coefficient (ICC). The ICC was 0,916 [−0,721,0.976] for the implementation subscale, 1.00 for the quality subscale, and 1.00 for the penetration rate subscale, and 0.983 [0,945;0,995] for the total fidelity scale.

## Discussion

This study presents the development and preliminary testing of the first fidelity scale to measure the structure, content, penetration rate and implementation of basic ACP. The scale is intended for use in geriatric hospital units, but with some modifications may also be suitable for health services in general, as its content is basic. This is of value since ACP is increasingly acknowledged as relevant to all individuals, and that it should be integrated into routine care, normalizing these discussions (39). The results should be interpreted with caution as there was little variation in measurements and validation of the scale should be further developed to include larger samples and samples over time. However, the results are stark and important and confirm that ACP requires a radical shift in its implementation to remain relevant (8, 9, 39).

The main findings of this study indicate that crucial structures necessary for implementing ACP in the units are absent, even in geriatric units that possess knowledge, tradition and expertise in communicating with older patients and next of kin. These include designated personnel responsible for coordinating and maintaining ACP practices, and commitment from leadership, training and supervision for staff, and having systems for identifying eligible patients, as well as documenting ACP to support quality improvement.

Regarding the quality of ACP, although some staff members were familiar with the principles, the majority of personnel lacked adequate knowledge about them. When ACP was practiced, several important components of the guidelines were often overlooked. These included assessing the patient's decision-making capacity, providing information about their health status tailored to the patient's preferences for information, and asking whether they had documented their healthcare wishes, such as through an advance directive or a living will which is more commonly used in Norway (1–3, 5). The observation that only the palliative care team in one of the included hospitals practiced ACP occasionally, also indicates a need for interdisciplinary collaboration and communication, and that ACP should not be limited to one team but integrated across all care teams involved in geriatric patient care, which is known from previous literature (9).

The results of this study also align with findings from other baseline data regarding the confidence levels and the extent of communication training on ACP among healthcare personnel in this context. A survey on self-assessed confidence in communication about future deterioration and medical care and the patient's own wishes towards end-of-life showed that this varies, and is limited among many geriatric health personnel in this setting (40). As part of the trial, we also aimed to investigate barriers and facilitators from within the Norwegian health care context more broadly (41). Known barriers that were echoed were the lack of knowledge and inadequate training that pose as a lack of communication skills and preparedness, as well as clinicians' discomfort and reluctance to talk about existential issues and dying and death (9, 24, 25, 42). This study highlights the need to build confidence and competence among healthcare personnel regarding ACP through knowledge, training, and supervision, as well as explicit prioritization from managers and policymakers, to embed ACP into the organizational culture.

Concerning the penetration rate, very few patients received an invitation to ACP, or were offered an ACP discussion that was also documented in the discharge note. This also suggests that policies and practices need to be revised to facilitate better integration of ACP documentation. Two previous reviews exploring the uptake of ACP for older patients including home dwelling elders in communities, describe that inconsistencies and variation in documentation act as barriers (43), and that healthcare professionals are often wary of documentation because they fear it may go against the patient's wishes (44). This study shows the absence of systems to document and communicate ACP across different levels, which is essential for ensuring continuity and supporting ACP as an ongoing process (1–3, 5, 9).

Subsequently, within the larger project, we used the knowledge gained from the baseline measurements to identify specific areas needing improvement and tailored our implementation strategy accordingly (45). For example, we offered practical training to units, facilitated by a communication expert, to help alleviate uncertainty around engaging in ACP. Additionally, we trained local staff and involved all personnel through a train-the-trainer model. We also developed and provided documentation templates for sharing among the units and are currently working towards agreeing on a common template to document ACP nationally.

Additionally, in the reports accompanying the fidelity measurements, the project team noted existing routines that could be used to improve the uptake, for instance, that nurses could be encouraged to identify eligible patients for physicians. Previous findings also suggests that a nurse-led pathway has been effective in increasing the frequency of ACP discussions in primary care settings (46).

## Strengths and weaknesses

The main strength of this study is the development of a new instrument that measures the implementation level of ACP in a standardized and structured manner. This allows for the assessment of all units using a single sample (45), and that areas that need improvement can be targeted, and monitored to track progress during an implementation program of ACP (45). It is also a strength that we have a better measure of the implementation level of ACP than in any previous study. Many of the items are also generic, which is a strength in terms of transferability to other parts of the health services.

A factor analysis cannot be performed at this stage, since the items have limited variation and thus undermine the possibility of using the models. The validation that was possible given the skewed data, a cross-sectional measurement, and that there are no other established measures of ACP practices, consisted of face and content validity, Cronbach's alpha for the subscales where this was possible, and a preliminary test of interrater reliability. In a follow-up study presenting fidelity assessment results at 7–10 months and 18 months, we will re-examine interrater agreement, conduct a CFA (Confirmatory Factor Analysis) and determine Cronbach's alpha for all subscales when we expect the level of implementation to be more varied across the units. We also plan to assess sensitivity to change, i.e., the ability of the scale to detect meaningful changes in ACP implementation after support (12) by comparing with other data in the larger project at the patient/next of kin level, clinical level and service level.

The gold standard in fidelity measurement is a site visit conducted by independent and experienced fidelity reviewers who observe the intervention, attend team meetings, interview staff and clients, and review medical records and program documents. A guiding principle for enhancing validity of ratings is triangulation, that is, seeking information from multiple sources to confirm ratings (12). However, this was not possible in this project due to the lack of time and resources, and the measurements are based solely on interviews with staff. Such an approach can make it feasible for units to adopt the fidelity scale to evaluate their own practice. To enhance the reliability and validity of ratings, future studies might consider including patient record reviews or direct observations, which would add depth and credibility to the assessments. Record review is time-consuming, especially when the item at issue is not consistently documented in a specific place such as ACP. In this study an important step was to set up a document type of its own so that ACP was consistently documented in the same type of document. A random selection of records that would include this type of document could save time in future iterations of this study, and a random selection would also guard against obvious sources of bias. Concerning the subscale on implementation, attending implementation team meetings could probably also be added as a source of data to confirm the accuracy of responses because ACP is a team-based EBP (involving the cooperation of different staff groups) (12), and the teams represent key staff involved in the implementation process. Reviewing program documents such as local procedures, electronic handbooks or manuals on ACP, information brochures and leaflets for patients could also increase the validity and reliability of the measurements (12), particularly on quality of practice. Including patients and next of kin as a data source could also increase the measurements on quality to confirm whether practice is in adherence with guidelines. Alongside the intervention, we will also conduct separate qualitative interviews with patients and next of kin, and collect questionnaires from staff, patients, and next of kin to assess service outcomes and patient outcomes and experiences. These data sources could also be used in further applications of the scale to triangulate findings on quality.

In terms of the representativeness of the sample, we only included acute geriatric units that have expertise in the care of older patients. This could pose as a problem of selection bias, and the scale should be tested in other contexts. In terms of external validity, these results do not necessarily reflect the situation in other wards that receive acutely ill home-dwelling older patients. Nevertheless, our preliminary measurements of ACP practices show that this is not well implemented even in wards that specialize in the care for older patients. Therefore, we have little reason to believe that ACP practices will be more systematic or of higher quality in other wards with older patient groups.

This scale is part of an ongoing national effort to improve ACP implementation and is intended for national or more local benchmarking after further validation, to establish standards for delivering ACP as a service to older patients. Evaluating whether improved fidelity gives better outcomes of the ACP intervention at the patient and service levels is the primary objective of the larger study (19). Future iterations of this scale may serve as a strategy to influence policy decisions by demonstrating the relationship between improved fidelity and program effectiveness. This is a way of providing evidence to support the allocation of resources to ACP programs that achieve high fidelity rates, particularly for more vulnerable patient groups. The scale assesses the structures necessary for integrating ACP into routine care, adherence to practice guidelines, as well as the penetration rate because dissemination of ACP is not simply about creating small exclusive programs for selected patients, but to make ACP easily accessible across the public health system. The scale can be used for internal quality improvement when using staff interviews, because it makes it feasible for units to use the scale to evaluate their own practices. We will also use the scale to do a research evaluation, including evaluating how closely the implementation of the intervention follows the original research protocol, checking whether all components were delivered as planned, and in which areas the research team should intervene to improve fidelity. Furthermore, we are continually refining and recalibrating the items based on our experiences with the scale, as well as evolving research and practices. This ensures that the scale remains relevant and reflective of best practices while also considering real-world conditions in clinical units.

## Conclusion

This study addresses a significant gap in the literature: the lack of a measurement tool to comprehensively assess the implementation of ACP to tackle the global challenge of insufficient implementation. This cross-sectional assessment conducted as a preliminary test of the scale i indicates that ACP is not routinely implemented in participating geriatric units, and that staff training, leadership involvement, and systematic follow-up are all lacking.

The scale is in its preliminary stages and further validation is necessary. Additional data are required to further establish its psychometric properties. We advise exercising caution in the use of the scale and in the interpretation of the results in this preliminary study.

The fidelity scale is intended for national or more local benchmarking to promote the uptake of ACP and improve the quality of health services for older patients. There is a gap between evidence and practice, and this scale can be used to identify and measure that gap, ultimately improving health services for older patients and their families.

## Data Availability

The raw data supporting the conclusions of this article will be made available by the authors, without undue reservation.
