# Supplementary material for: Measuring the level of implementation of advance care planning – a fidelity-based cross-sectional study
Source: Front Health Serv. 2025 Oct 29;5:1629242. doi: 10.3389/frhs.2025.1629242 (PMC12605388; doi:10.3389/frhs.2025.1629242)
Supplement: Supplementary file 1 [file Datasheet1.docx]

| **Items 1-4 – Implementation measures in the start-up phase** | | | | | | | **Changes made and why** |
| --- | --- | --- | --- | --- | --- | --- | --- |
| **1^[[1]](#footnote-2)^** | At least one **kick off** at the unit regarding the implementation of advance care planning has been held with important staff. That is:  a) **Head** of the department  b) **Physicians**  c) **Nurses**  d) Other associated **healthcare workers**  e) **Key workers** among all staff | No (no meetings). | At least one of the key staff groups has met. | Some of the key staff groups have met. | Most of the key staff groups have met. | All of the key staff groups have met. | The term "meeting" was changed to "kick off" to more accurately reflect the initiative's purpose, i.e. that staff come together to discuss the action plan and to ensure everyone is aligned and understands the goals moving forward. We kept the emphasis on key staff because ACP is a team-based EBP. We have experienced in the project and recommend for further iterations of the scale that also regular meetings should be held to maintain implementation, for instance every 6 months. The item could therefore be removed or changed into including this. In the project we decided to keep it as is and scored this as a "kick off” since the start of the project. One should also discuss whether having separate meetings within key staff groups, for example for nurses and physicians also should count as a full score. |
| **2** | An **action plan** for implementing advance care planning in the unit has been written and key staff groups (see item 1) are aware of it. | Nothing has been done. | An action plan exists, but staff are not aware of it. | An action plan exists, and at least one staff group is aware of it. | An action plan exists, and at least two staff groups are aware of it. | An action plan exists, and all staff groups are aware of it. | We underscored that the action plan should be a living document and known to staff, i.e. not only be a written document. We have also established a timeframe, i.e. that the plan should be revisited at least every six months. This to ensure that it is regularly updated and not merely presented passively to staff at one point in time with no follow up. |
| **3** | **Program philosophy**: The unit has a clear understanding of advance care planning in adherence to guidelines^^[[2]](#footnote-3)^^.  If staff spontaneously demonstrate this without prompting, it gives an extra point.  The scoring is based on these five groups:  a) **Head** of the department  b) **Coordinator**  c) **Physicians**  d) **Nurses**  e) **Other healthcare personnel** involved in advance care planning | None of the groups demonstrate a clear understanding of the program philosophy  OR  All groups show lack of understanding in numerous significant areas. | At least one of the five groups demonstrates a clear understanding of the program philosophy  OR  All groups show lack of understanding of some significant areas. | Some of the groups demonstrate a clear understanding of the program philosophy  OR  The groups generally concur with the program philosophy, but show lack of understanding. of one major area | Most of the groups demonstrate a clear understanding of the program philosophy  OR  The groups are generally in agreement with the program philosophy but show lack of understanding of one or two minor areas. | All groups show a clear understanding and commitment/engagement concerning the program philosophy for advance care planning conversations. | The key principles of advance care planning, as outlined in the guidelines, were integrated into the scale itself for clarity when scoring. |
| **4** | **Coordinator:**  a) The unit has identified a coordinator for advance care planning (the responsibility may be shared by more than one person).  b) The definition of the role and responsibilities are clear and explicit.  c) The coordinator receives training in advance care planning conversations and has opportunities for supervision (e.g., annually).  d) The coordinator keeps track of the advance care planning conversations that have been conducted in the unit. | 0 criteria | 1 criterion | 2 criteria | 3 criteria | 4 criteria | In four of the units the leader took on the task in the absence of a coordinator, and we discussed whether we should we give credit for it. We decided not to and leave the scale as such. For later iterations of the scale, one could possibly adjust points b, c, d to also include having designated time and to include whether the work is done and the quality of the work. |
| **5** | **Training and supervision**  The unit accommodates annual training for both existing and newly hired staff in the following areas (if annual training is not offered, the criteria are not met. Training means lectures for all and practical training for those who will participate in advance care planning conversations):  a) Instruction in key principles of the program philosophy (see item 3; not all points must be covered in detail)  b) Patients’ and relatives’ legal rights and roles relevant to advance care planning conversations, and the healthcare service’s obligations toward them  c) Use of conversation guides or pocket cards on how to conduct advance care planning conversations  d) Simulation/practical exercises  e) An overview over barriers and facilitators for advance care planning in hospitals  f) Documentation and interprofessional collaboration  g) Staff must have access to supervision (e.g., from the coordinator, trained staff, reflection groups, or clinical ethics committees in the event of ethical dilemmas). | 0 criteria | 1-2 criteria | 3-4 criteria | 5-6 criteria | 7 criteria | We continually encountered the issue that the item was somewhat challenging to score because it is perhaps too strict to have 'annual’ training. We decided to score it as such, but for future iterations of the scale, one should discuss as part of face validity whether this is something that can be implemented in practice or fits with how they otherwise structure training. We also specified in the item that teaching should encompass the key principles outlined in the guidelines; however, we concluded that not all principles were necessary for achieving a full score, as we found that covering all of them would not set a realistic threshold, even for dedicated health personnel. Additionally, we included 'pocket cards' since they were developed during the implementation of the program as part of the toolkit. |
| **Items 6-9 – Identification of patients and penetration rate** | | | | | | |  |
| **6** | **Systematic screening to identify eligible patients.**  A system exists to identify patients suitable for advance care planning conversations.  This system is implemented as part of routine practice.  A system is in place for patients currently deemed ineligible, to ensure they may be offered the opportunity later (for example, noting in discharge summaries that certain important decisions may need to be made going forward).  A routine exists to invite relatives, particularly when the patient lacks capacity to consent to an advance care planning conversation. | 0 criteria | 1 criterion | 2 criteria | 3 criteria | 4 criteria | The item was not changed, but for d) we discussed and agreed that scores should be given when the unit had an established routine for inviting patients as well as relatives, also when the patient lacked decision-making capacity (for instance in one unit, the clinicians always invited relatives in, but did not have any systematic routine for including patients). The rationale was that there should be a systematic routine to include both parties when this is the patient’s preference, and that it is also a way of giving patients who lack capacity to consent an opportunity to have ACP in line with practice guidelines for patients with dementia.  For future iterations of the scale, point a) could be an 'entry criterion' for receiving points for the other items, particularly b) and d), and one could more accurately define what counts as a system or routine. |
| **7** | **Percentage of patients invited to advance care planning** in the past six months.  Proportion of patients and or relatives who, prior to discharge, have been invited to an advance care planning conversation out of the total number of geriatric patients admitted to the unit. A verbal invitation is sufficient. | 0-20% | 21-40% | 41-60% | 61-80% | 81-100% | A timeframe of six months was added to establish a cut-off date, to get more reliable measurements. |
| **8** | **Percentage of admitted patients** **who have** **received advance care planning** prior to discharge in the last six months.  This can be together with relatives, or with relative alone. | 0-20% | 21-40% | 41-60% | 61-80% | 81-100% | This project adopted an approach that encouraged the involvement of relatives, permitting conversations with them only when patients were unable or chose not to participate in line with the consensus definition for patients with dementia. Therefore, we have included in the item that advance care planning could be conducted solely with the relatives. |
| **9** | **Assessment of the patient’s capacity to consent to an advance care planning conversation.**  Percentage of completed advance care planning conversations in the past six months (possibly with patient or relatives alone) in which the patient’s capacity to consent was evaluated. If this evaluation takes place during the conversation, it still counts as a full score. | 0-20% | 21-40% | 41-60% | 61-80% | 81-100% | We included in the item that advance care planning could be conducted solely with the relatives for the same reasons as explained above. We also added the time frame of six months to give more reliable measurements. |
| **Items 10–17 – Quality of the advance care planning conversation**  The score should be based on the advance care planning conversations that have been conducted in the past six months  These criteria address adherence to recommended guidelines for conducting advance care planning conversations. Elements 10–16 are graded based on the proportion of conducted advance care planning conversations meeting specific standards. The scoring should emphasize responses from the advance care planning coordinator, resource staff, and healthcare professionals at the unit who conduct these conversations, as well as any relevant procedures/written materials. | | | | | | |  |
| **10** | **Percentage of completed advance care planning conversations where the patient received both a** **written and verbal invitation.** | 0-20% | 21-40% | 41-60% | 61-80% | 81-100% | Elements 10-16 were changed from being based on the number of sources demonstrating a clear structure in adherence with the recommendations to being scored as percentages of the advance care conversations conducted in the last six months. However, the scoring would still emphasize the responses from the coordinator, resource staff, and healthcare personnel conducting advance care planning, and we also included any procedures/written materials. Additionally, under element 16, we changed "can" to "will" in terms of communication onwards, because once consenting to ACP communicating ACP is something that will be done and is an essential part of ACP. |

| **11** | **Percentage of completed advance care planning conversations in which the patient’s** **relatives were involved in accordance with the patient’s wish**. | 0-20% | 21-40% | 41-60% | 61-80% | 81-100% | We did not change the item but noted that several units requested the possibility to involve relatives after the ACP conversation if they were not present at the moment, by calling them afterwards. The question assumes that the patient is capable of giving consent. Therefore, we scored the involvement of relatives before or after the conversation as equivalent—primarily because we did not ask in a sufficiently presise manner and did not have detailed enough notes to differentiate this retrospectively. |
| --- | --- | --- | --- | --- | --- | --- | --- |
| **12** | **Percentage of completed advance care planning conversations in which the patient** **received information about their health status, prognosis, and key healthcare decisions** likely to arise in the near future, adapted to the patient’s preference for information. | 0-20% | 21-40% | 41-60% | 61-80% | 81-100% | See item 10. Changed from being based on the number of sources demonstrating a clear structure to percentages of the advance care conversations conducted in the last six months in which the criteria are met. |
| **13** | **Percentage of completed advance care planning conversations in which what matters most to the patient was addressed.**  For example, what supports the patient’s quality of life, considerations about the future, the final phase of life, or death. | 0-20% | 21-40% | 41-60% | 61-80% | 81-100% | Same as over. |
| **14** | **Percentage of completed advance care planning conversations in which it was** **opened up to talk about future healthcare preferences**.  This may include care or different types of life-prolonging treatment. | 0-20% | 21-40% | 41-60% | 61-80% | 81-100% | Same as over. |
| **15** | **Percentage of completed advance care planning conversations in which the patient was asked if they had documented their wishes** for future healthcare.  This may include a living will or advance directive. | 0-20% | 21-40% | 41-60% | 61-80% | 81-100% | Same as over. |
| **16** | **Percentage of completed advance care planning conversations in which it was** **explained that the content of the conversation will be communicated onwards**.  This may be to the patient’s general practitioner, a nursing home physician, or other relevant healthcare personnel. | 0-20% | 21-40% | 41-60% | 61-80% | 81-100% | Same as over. |
| **17** | **Summary and evaluation of the advance care planning conversation**  (Each criterion is counted only if it applies to more than 50% of the conversations):  a) A summary was made during the conversation to check for mutual understanding of what had been brought up.  b) The patient and relatives were asked how they experienced discussing these issues.  c) The patient/relatives were asked whether there were other preferences that had not been addressed in the conversation.  d) The patient was informed that he or she could request a new advance care planning conversation and revise the preferences. | 0 criteria | 1 criterion | 2 criteria | 3 criteria | 4 criteria | 17 c) was changed from ‘provide feedback’ to ‘ask whether there were other preferences that had not been addressed in the conversation’. The rationale was that this is more in line with practice guidelines, and that providing feedback was part of the implementation support subscale and is already covered in 22 b). |
| **Items 18-21 – Documentation and coordination** | | | | | | |  |
| **18** | **Percentage of patients admitted to the unit in the last six months for whom it is** **checked for prior advance care planning in the patient record**.  This could be, for example, in the referral or notes from previous admissions. | 0-20% | 21-40% | 41-60% | 61-80% | 81-100% | No changes made. |
| **19** | **Quality of documentation of the current ACP**  The score is based on how many of the following criteria are met among advance care planning conversations conducted in the past six months (each criterion only counts if it applies to more than 50% of the conversations held):  Documentation of:  a) Who participated is documented  b) Evaluation of the patient’s capacity to consent to participate is documented  c) Main topics brought up in the conversation are documented  d) Documentation is visible and accessible to other staff  e) The patient and/or relatives have received a copy of the content for their perusal. | 0 criteria | 1 criterion | 2 criteria | 3 criteria | 4-5 criteria | 19 e) was changed to ‘and/ or’ because all patients should receive a copy, but there is not necessarily a separate copy for relatives unless the patient gives consent. In cases where the relatives have the ACP conversation as a representative of the patient who has lost capacity, only a copy for the relatives for perusal would be appropriate. |
| **20** | **Percentage of patients for whom** **advance care planning was included in the discharge summary** **among those who have had a conversation** (in the department in the past six months). | 0-20% | 21-40% | 41-60% | 61-80% | 81-100% | No changes made. |
| **21** | **Percentage of patients whose** **advance care planning was included in the discharge summary, out of the total number of admitted patients** (in the department during the past six months). | 0-20% | 21-40% | 41-60% | 61-80% | 81-100% |  |
| **Item 22 Ongoing implementation measures** | | | | | | |  |
| **22** | **Continuous implementation measures**  Scored based on how many of the following measures are implemented:   1. An implementation team that includes the head of the department has been established and is actively working to improve the implementation of advance care planning in the department (e.g., regular meetings). 2. The implementation team has routines for gathering feedback on how patients and relatives felt they were involved. 3. Regular evaluations of the implementation process are conducted, and the results are actively used to guide quality improvement. 4. Regular evaluations are conducted regarding how patients and relatives experience their involvement in the department, and the results are actively used to guide quality improvement. 5. The implementation team has shared an overview of common barriers and facilitators to advance care planning in hospitals with staff. | 0-1 criteria | 2 criteria | 3 criteria | 4 criteria | 5 criteria | The item was not changed, but an important discussion was whether we should give credit for implementation teams 'on paper'. We decided not to (this was the case for three units in the study). The rationale was that inactive teams are unlikely to implement changes effectively. Recognizing only active teams also encourages commitment. We experienced in the project that it is essential to hold teams responsible for maintaining their engagement. Another rationale is that resources are often limited, and it is more justifiable to allocate support to those teams that are actively working and making progress.  Focusing on active teams also prioritizes quality initiatives and  provides data that can be used to measure success, making it easier to assess the program’s effectiveness.  Further iterations of the scale could include 'active' or 'functioning', or ‘having had at least one meeting in the last 6 months’. |

1. Items marked in blue represent implementation measures, items marked in green indicate quality of practice, and items in purple reflect penetration rates. [↑](#footnote-ref-2)
2. Key principles of advance care planning (ACP) (please see Additional file 4 for an overview of the evidence base for the key items in the scale):

   • Ensuring patient autonomy and involvement: The purpose of ACP is to safeguard the patient’s right to autonomy and shared decision-making. The topics and questions should be tailored to the patient’s preferences for a preparatory discussion. The guiding question should be, “What is important to you?”.

   • Voluntary participation: The conversation is voluntary, and the patient’s decision-making capacity should be assessed before engaging in such conversations. Additionally, the role of relatives should be considered in accordance with the patient’s decision-making capacity and wishes.

   • Addressing the patient’s preferences for information and involvement: The discussion should explore the extent to which the patient wishes to receive information about and participate in decisions regarding their health. Future preferences for medical treatment and care, including end-of-life considerations, should be discussed if the patient desires.

   • Incorporating values and beliefs: ACP may also include conversations about values, personal beliefs, and preferences regarding end-of-life care, should the patient wish to engage in such conversations. These conversations might occur through so-called “golden moments,” which refer to informal, patient-initiated conversations with healthcare personnel during daily activities, such as personal care, as part of a whole-ward approach.

   • Involvement of all relevant parties: All involved parties should participate in the process, including the patient, healthcare personnel, and, where appropriate, the patient’s relatives, provided the patient consents or lacks decision-making capacity.

   • Continuous process: Advance care planning is an ongoing process that evolves over time.

   • Documentation of conversations: It is essential to document the conversations to ensure their utility in the future, including both internal and external communication (see also items 18, 19, and 20).

   • Inclusion of advance directives: ACP conversations may result in the creation of an advance directive or living will.

   • Timing considerations: The timing of ACP conversations is crucial and often challenging. An individualized approach is recommended. While such conversations can occur at any time if the patient is ready, they become particularly relevant in cases of advanced age, chronic illness with the potential for deterioration, or situations where the patient’s ability to participate may decline. However, it is essential to ensure the conversations do not occur too late. ACP is also pertinent for elderly patients admitted acutely to hospitals, especially those who are frail, exhibit early cognitive decline, or have other serious chronic illnesses. [↑](#footnote-ref-3)
