# Supplementary material for: Measuring the level of implementation of advance care planning – a fidelity-based cross-sectional study
Source: Front Health Serv. 2025 Oct 29;5:1629242. doi: 10.3389/frhs.2025.1629242 (PMC12605388; doi:10.3389/frhs.2025.1629242)
Supplement: Supplementary file 2 [file Table1.docx]

**Additional file 2: Key items in total fidelity scale for ACP including evidence base**

| **Item description** | **Item number** | **Recommendations** | **Key references** |
| --- | --- | --- | --- |
| ***Implementation subscale*** |  | ***The unit should*** |  |
| Meeting | 1 | Organize at least one meeting about the implementation of ACP with key staff. | (1) |
| Action plan | 2 | Make an action plan that is known to the staff. | (1, 2) |
| Program philosophy | 3 | Be committed to a clearly articulated program philosophy consistent with the evidence-based practice (EBP) where the entire ward is involved. | (2-6) |
| Coordinator | 4 | Appoint a coordinator to help implement and sustain the practice. Relevant tasks will be providing internal training and supervision to local personnel and overseeing implementation efforts. | (1, 2, 6) |
| Training and supervision | 5 | Provide all clinicians with basic competence and skills, to ensure that ACP becomes one of the cornerstones of treatment. | (1, 6) |
| Systematic identification of eligible patients | 6 | Have a system to identify eligible patients for ACP. | (1, 6, 7) |
| Continuous implementation measures | 22 | Establish an implementation team to organise and supervise the implementation process. | (2, 6) |
| ***Quality subscale*** |  | ***Clinicians in the unit should*** |  |
| Assessing decision-making capacity. | 7 | Assess the patient’s decision-making capacity for having an ACP-discussion as part of the identification of eligible patients. If the patient lacks decision-making capacity, the conversation should strive to include the next of kin to support the patient and adjust the conversation to the patient’s level of cognition to support the patient’s autonomy. | (7-11) |
| Verbal and written invitation. | 10 | Give verbal and preferably written invitations to patient and/or relatives. The invitation should include that the conversation is voluntary, the purpose and the content of the conversation, and who should participate. | (7, 9) |
| Level of involvement | 11 | Recognize the person’s level of readiness and tailor information and the discussion to the person’s willingness to engage, as well as recognize the patient’s preferences about who should be involved. | (3, 4, 8, 10, 11) |
| Provide information | 12 | Provide the patient with information about their current health status and what choices they may face in the future, tailored to how much information the patient is seeking. This can also be an opportunity to clear up eventual misconceptions about their health. | (3, 8, 10, 12) |
| What is important to the patient | 13 | Try to get an understanding of the patient’s values and beliefs, for example by asking what is important to the patient. | (3, 4, 7-10) |
| Elicit preferences regarding future and current treatment and care | 14 | Open up for talking about different care and treatment options. This can be issues like inpatient hospitalization, the utilization of “life support” or CPR. | (3, 4, 8, 12) |
| Previous documentation of patient’s values, goals and preferences. | 15 | Check for previous documentation of patient’s values, goals and preferences (for instance in a living will). | (3, 8, 9) |
| Permission to pass on information to other health personnel | 16 | Ask for permission to disseminate the content of the ACP-discussion to others who would benefit from the information, for instance family members, family doctor or others. | (8, 9) |
| Summing up and evaluating the ACP-discussion | 17 | Sum up and evaluate the ACP-discussion. This includes the following four points: summing up and checking if there is a common understanding of the information given in the ACP-discussion, asking the patient/ and next of kin how they experienced having the discussion, inviting them to give feedback about needs that weren’t met in the discussion, and informing the patient/ next of kin that the patient can revise his or her care plan at any time. | (3, 4, 8, 9) |
| Quality of documentation | 19 | Document ACP-conversations in the medical record of the patient. This includes the following five points: who participated, the assessment of decision-making capacity, the most important content of the ACP-discussion, that documentation is accessible for other health personnel, and that patient and next of kin were given a copy for their perusal. | (3, 4, 7-10) |

| Dissemination of ACP in discharge summary | 20 | Disseminate ACP in the discharge summary of the patient. | (6-8) |
| --- | --- | --- | --- |
| ***Penetration rate subscale*** |  |  |  |
| Proportion invited to ACP | 8 | Maximize the proportion of patients invited to ACP among the total number of admitted patients. | (2, 7, 9) |
| Proportion given ACP | 9 | Maximize the proportion of patients given ACP among the total number of admitted patients. | (2, 6) |
| Proportion of patients where the unit checks for previous documentation ACP | 18 | Check in the patient record for previous documentation of ACP/AD for newly admitted patients. | (3, 8, 9) |
| Proportion of patients where ACP figures in discharge summary | 21 | Maximize the number of patients who have documented ACP in their discharge summary. | (6) |

**References**

1. Reynolds J, Croft S. How to implement the Gold Standards Framework to ensure continuity of care. Nurs Times. 2010;106(32):10-3.

2. Evaluating Your Program. Rockville, MD: Center for Mental Health Services,

Substance Abuse and Mental Health Services Administration: U.S. Department of Health and Human Services; 2009.

3. Rietjens JAC, Sudore RL, Connolly M, van Delden JJ, Drickamer MA, Droger M, et al. Definition and recommendations for advance care planning: an international consensus supported by the European Association for Palliative Care. Lancet Oncol. 2017;18(9):e543-e51.

4. Sudore RL, Lum HD, You JJ, Hanson LC, Meier DE, Pantilat SZ, et al. Defining Advance Care Planning for Adults: A Consensus Definition From a Multidisciplinary Delphi Panel. J Pain Symptom Manage. 2017;53(5):821-32.e1.

5. Sævareid. Advance care planning in nursing homes: a mixed method study of a complex intervention using a whole-ward approach University of Oslo; 2019.

6. Hestmark L, Heiervang KS, Pedersen R, Hansson KM, Ruud T, Romøren M. Family involvement practices for persons with psychotic disorders in community mental health centres - a cross-sectional fidelity-based study. BMC Psychiatry. 2021;21(1):285.

7. Ahmed M. Procedure for advance care planning. Oslo University Hospital; 2020.

8. Detering KM, Silveira MJ. Facilitating advance care planning discussion (UpToDate) [cited 2020 1.2.2020]. Available from: <https://www.uptodate.com/contents/advance-care-planning-and-advance-directives?search=advance%20care%20planning&source=search_result&selectedTitle=1~150&usage_type=default&display_rank=1#H2094995>

9. Thoresen L LL, Sævareid TJL, Gjerberg E, Førde R, Pedersen R. Guide. Advance care planning (ACP) - planning for future health care and end-of-life care together University of Oslo2015 [cited 2022 03.08.2022]. Available from: <https://www.med.uio.no/helsam/english/research/projects/end-of-life-communication-in-nursing-homes/acp-guide070617.pdf>.

10. National recommendations for advance care planning. Oslo: Norwegian Directorate of Health; 2023.

11. van der Steen JT, Nakanishi M, Van den Block L, Di Giulio P, Gonella S, in der Schmitten J, et al. Consensus definition of advance care planning in dementia: A 33-country Delphi study. Alzheimer's & Dementia. 2024;20(2):1309-20.

12. Gleeson A, Noble S, Mann M. Advance care planning for home health staff: a systematic review. BMJ Support Palliat Care. 2021;11(2):209-16.
